# Perioperative hypersensitivity reactions: a retrospective study (2018–2023) in a Lebanese tertiary clinic

**DOI:** 10.3389/fphar.2026.1767932

**Published:** 2026-03-25

**Authors:** Diane Antonios, Guitta Badran, Viviane Chalhoub, Luc De Chaisemartin, Pascale Roland Nicaise, Sylvie Chollet-Martin, Marc Pallardy, Hayat Azouri, Carla Irani

**Affiliations:** 1 Laboratory of Toxicology, Faculty of Pharmacy, Saint Joseph University of Beirut, Beirut, Lebanon; 2 Department of Anaesthesia, Critical Care and Pain Management, University Medical Center Hôtel-Dieu de France Hospital, Faculty of Medicine, Saint Joseph University of Beirut, Beirut, Lebanon; 3 Immunology Department, Assistance Publique-Hôpitaux de Paris (APHP), Bichat Hospital, Paris, France; 4 Université Paris Cité, INSERM UMR 1149, Centre de Recherche de l’Inflammation, Innalung Team, Faculté Bichat, Paris, France; 5 Université Paris-Saclay, INSERM, Inflammation Microbiome Immunosurveillance, Orsay, France; 6 Internal Medicine and Clinical Immunology, University Medical Center Hôtel-Dieu de France Hospital, Faculty of Medicine, Saint Joseph University of Beirut, Beirut, Lebanon

**Keywords:** epidemiology, Lebanon, neuromuscular blocking agents, opioids, perioperative hypersensitivity

## Abstract

**Background:**

Perioperative hypersensitivity (POH) reactions are of significant concern for anesthesiologists and allergologists, often leading to surgery delays, extended hospital stays, and increased morbidity and mortality. The main objective of this study was to provide an overview of POH reactions in Lebanon and their evaluation.

**Methods:**

A 5-year retrospective review was conducted at a tertiary allergy clinic in Lebanon, involving patients with a history of POH reactions or preoperative evaluations due to atopy or known drug, respiratory and/or food allergies. All patients underwent skin prick tests (SPTs) for several perioperative agents.

**Results:**

A total of 255 patients were included in this study. Among them, 124 patients (48.6%) were referred to the clinic with a history of POH reactions, mostly immediate, with symptoms ranging from mucocutaneous manifestations to cardiopulmonary arrest. SPTs were conducted for all 255 patients, focusing on opioids, neuromuscular blocking agents (NMBAs), hypnotics, local anesthetics, latex and patent blue. Of these, 97.3% (n = 248) demonstrated a positive reaction to at least one substance. The most frequent sensitizations were to morphine (59.7%, n = 148), rocuronium (54%, n = 134), and latex (48%, n = 119), while sensitizations to hypnotics (19%, n = 47) and local anesthetics (8.5%, n = 21) were less common. We assessed co-sensitization within the same drug class, identifying rocuronium and cisatracurium as the NMBAs with the highest co-sensitization, while morphine and pethidine showed the highest co-sensitization among opioids. Among the 124 patients with a history of POH reactions, SPTs results identified the causative agent, primarily morphine, fentanyl or rocuronium, in 38 patients.

**Conclusion:**

This study provides valuable insights into the clinical characteristics of POH reactions in Lebanon and highlights the role of SPTs in identifying causative agents thus allowing to suggest alternative perioperative options.

## Introduction

Perioperative hypersensitivity (POH) reactions are generally immediate, rare and unpredictable reactions (Moreau et al., 2023). Their incidence varies considerably, from 1 per 353 to 1 per 18,600 operations, due to the different reporting approaches, the heterogeneity of studies and the international diversity of clinical practices (Beyaz et al., 2022). The overall incidence has therefore been underestimated and set at around 1 per 10,000 operations (Pitlick and Volcheck, 2022; Pirson et al., 2018). Mortality rates for perioperative anaphylaxis vary by country, with the most recent data showing an incidence of 1 per 313,000 procedures in Europe and 1 per 191,652 procedures in the United States (Tacquard et al., 2023; Kosciuczuk and Knapp, 2021).

Any substance administered or in direct contact with the patient in the perioperative setting is likely to cause hypersensitivity reactions, including neuromuscular blocking agents (NMBAs), opioids, hypnotics, local anesthetics, latex, patent blue, antibiotics, antiseptics, sugammadex, and others (Garvey, 2016).

Mast cells and basophils are the key effector cells of POH and anaphylaxis. Degranulation of these cells can be triggered by allergic or non-allergic mechanisms (Sabato et al., 2019). Allergic reactions occur when a specific adaptive immune response is involved. Specific activation of sensitized mast cells and basophils occurs through allergen-driven cross-linking of IgE receptors, leading to degranulation via the classic IgE-mediated mechanism (Pitlick and Volcheck, 2022; Ebo et al., 2019). Anaphylaxis can also result from non-IgE mechanisms, including the IgG–neutrophil pathway, implicated in drugs such as NMBAs (Bruhns and Chollet-Martin, 2021; Jönsson et al., 2019), and direct mast cell activation via Mas-related G protein–coupled receptor X2 (MRGPRX2) by drugs such as opioids and NMBAs. Nonspecific activation can also be mediated by other pathways such as the activation of the complement system, associated with the kallikrein-kinin system, and the cyclooxygenase-1 pathway (Pitlick and Volcheck, 2022; Bruhns and Chollet-Martin, 2021; Jönsson et al., 2019; Volcheck et al., 2023).

Activation of mast cells triggers the release of preformed inflammatory mediators such as histamine and tryptase, and other mediators such as platelet-activating factor (PAF), prostaglandins, interleukins, bradykinin and leukotrienes (Salik and Hernandez, 2019). In consequence, clinical manifestations extend from a mucocutaneous reaction to severe anaphylaxis, marked by multivisceral lesions that can lead to cardiovascular and/or pulmonary collapse, or even death (Moreau et al., 2023). The most quoted grading system has been developed by Ring and Messmer, which includes four grades: Grade 1 (mild) involves skin symptoms, Grade 2 (moderate) includes multi-organ involvement with hypotension and bronchial hyperreactivity, Grade 3 (life-threatening) features severe cardiovascular and respiratory impairment, and Grade 4 (arrest) corresponds to circulatory or respiratory arrest (Dewachter and Savic, 2019; Ring and Messmer, 1977).

Etiologic diagnosis constitutes a challenge for anesthesiologists and allergologists due to the numerous differential diagnoses, unusual clinical manifestations, and simultaneous administration of a range of perioperative agents (Thanachit et al., 2024). Thus, all the patients with a suspected POH reaction should be referred for allergological evaluation to establish a strategy for prevention of these reactions during subsequent anesthesia (Volcheck et al., 2023). This investigation includes serum tryptase levels at the time of reaction and skin tests 3 weeks later, but may also involve specific IgE assays, basophil activation tests (BAT) and provocation tests (Volcheck and Mertes, 2014). Ideally, the causal agent of these accidents should be identified, but unfortunately this will not be possible in all cases (Tacquard et al., 2023).

To the best of our knowledge, there are very few published studies assessing POH from the Middle East that include a substantial patient population and present novel findings (Irani, 2014; Dagher et al., 2024; Yegin et al., 2025; Al-Ahmad et al., 2021). In the current study, we conducted a five-year retrospective study in a tertiary allergy clinic to provide an overview of POH reactions in Lebanon, as well as the results of skin prick tests (SPTs) performed to highlight the most causative agents.

## Methods

### Study design

We conducted a 5-year retrospective review (March 2018 to March 2023) of electronically archived medical records for patients who underwent SPTs for perioperative agents. These patients were referred to the outpatient allergy clinic at the University Medical Center Hôtel-Dieu de France (HDF) in Beirut, a tertiary-care allergy center in Lebanon. Referrals originated from HDF as well as other hospitals across Lebanon. Records were archived on an electronic International Organization for Standardization–certified telemedicine platform, TrakMD a certified digital electronic healthcare platform (ISO-certified 27001:2013). Our study was approved by the HDF ethical committee (case number CEHDF 1598).

### Demographic information, study variables, and analysis

**FIGURE 1:**
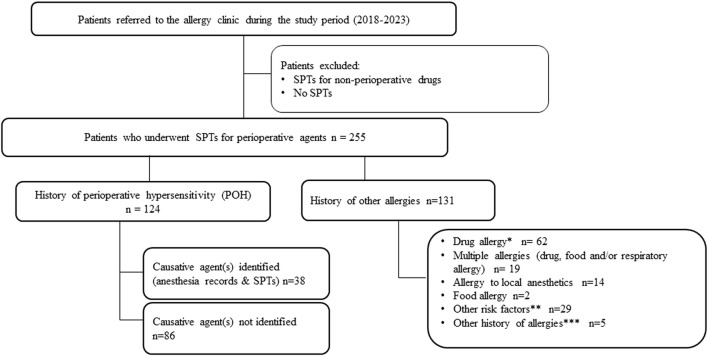
Study design and patients’ selection ^*^Penicillins, cephalosporins, sulfonamides, fluoroquinolones, macrolides, chloramphenicol, nitroimidazole, non-steroidal anti-inflammatory drugs, hyoscine butylbromide, methylprednisolone, acetaminophen, aspirin, rabeprazole, iron, contrast agents. **Cutaneous (Angioœdema, Dermatitis, Urticaria) or respiratory (Allergic rhinitis, Asthma) symptoms, family history of drugs allergy or anaphylaxis to perioperative agents ***Latex, botulinum toxin, nylon or adhesives.

Data collection was performed anonymously by assigning an identification number to each patient and included sex, age, date, reason for consultation and medical history. The major reasons for consultation were a history of POH reaction or the presence of risk factors requiring evaluation by SPTs before scheduled anesthesia. Patients presented with risk factors such as a history of drug allergy, a history of other or multiple allergies (e.g., drug, food, and/or respiratory), a history of allergy to local anesthetics, a family history of allergy, and various cutaneous and respiratory signs. The study design, patient selection, and subgroup allocation are detailed in Figure 1. All patient files included in our study were from patients who had undergone SPTs for a range of agents administered perioperatively. Following consultation with the allergist, SPTs were performed for some or all of these molecules: opioids (morphine, pethidine, sufentanil, fentanyl, remifentanil, alfentanil, oxycodone, and tramadol), NMBAs (rocuronium, cisatracurium, atracurium and succinylcholine), hypnotics (midazolam, ketamine, propofol, thiopental and etomidate), local anesthetics (lidocaine, bupivacaine, ropivacaine and mepivacaine) and other agents such as latex, and patent blue. The results of SPTs performed on all patients and the clinical manifestations for patients with a history of POH reactions were collected. Furthermore, circulating tryptase at the time of reaction and total IgE levels were measured in some patients, when appropriate samples were available.

The descriptive study was carried out using Excel 2016 (Microsoft Office). Quantitative and qualitative variables were expressed as medians and percentages, respectively. To calculate the percentage of sensitization for all patients evaluated by SPTs, for each molecule in the studied population, the following formula was used:

$$\frac{\text{Number}\;\text{of}\;\text{positive}\;\text{results}\;\text{for}\;\text{the}\;\text{molecule}\;\text{obtained}\;\text{through}\;\text{SPTs}\;}{\text{Number}\;\text{of}\;\text{patients}\;\text{with}\;a\;\text{positive}\;\text{SPTs}}x100$$

### Skin prick test

SPTs were performed according to American Academy of Allergy, Asthma and Immunology (AAAAI) guidelines (Supplementary Table S1). All molecules were provided by the pharmacy or the operating room of the HDF hospital. The test was performed via a prick to the skin of the forearm with a Stallerpoint lancet (Stallergenes Greer, Baar, Switzerland). Results were evaluated after 15 min under close supervision. Patients were instructed to discontinue all antihistamine medications 5 days before testing. SPTs results were considered positive if a wheal and flare reaction of ≥3 mm was observed, compared to histamine and saline, which served as positive and negative controls, respectively. Notably, all the tests were performed by the same operator, ensuring consistency and reproducibility.

## Results

All patients’ medical records from March 2018 to March 2023 were retrospectively analyzed, and 255 patients met the study criteria.

Table 1 presents the demographic characteristics of patients consulting an allergologist due to a history of POH reactions or the presence of risk factors requiring evaluation with SPTs prior to scheduled anesthesia. Patients were predominantly female (84.0%, n = 214), with a female-to-male ratio of 5.22:1. The median age was 42 years, ranging from 1 to 75 years Table 1 shows that 48.6% (n = 124) of consultations were attributed to a history of POH reactions, with the vast majority (n = 123, 99%) involving immediate reactions. Additionally, 24.3% (n = 62) of consultations were related to a history of drug allergies, including allergies to antibiotics (penicillins, cephalosporins, sulfonamides, fluoroquinolones, macrolides, chloramphenicol, or nitroimidazole), non-steroidal anti-inflammatory drugs (NSAIDs), hyoscine butylbromide, methylprednisolone, acetaminophen, rabeprazole, iron, and/or contrast agents. Moreover, 7.5% (n = 19) of patients had a history of multiple allergies (drug, food, and/or respiratory), 5.5% (n = 14) reported a history of local anesthetic allergies in dental or pre-Botox contexts, and 0.8% (n = 2) had a history of food allergies. Other risk factors were identified: 7.1% (n = 18) of consultations were due to cutaneous signs such as angioedema, dermatitis, and urticaria, while 2.7% (n = 7) were due to respiratory signs, including allergic rhinitis and asthma. Additionally, 1.6% (n = 4) of consultations were related to a family history of allergies, with 1.2% (n = 3) involving a family member who had experienced anaphylaxis to agents used in perioperative care. Furthermore, 2% (n = 5) of patients had a history of allergies to latex, botulinum toxin, nylon, or adhesives.

**TABLE 1:** Demographic characteristics of patients performing skin prick tests for perioperative reactions (March 2018-March 2023).

Total number of patients performing SPTs	255	
Gender	n	(%)
Female	214	84.0
Male	41	16.0
Age range (median)	1–75 years	(42 years)
Reason for consultation	n	(%)
History of perioperative hypersensitivity reactions	124	48.6
Immediate	123	​
Delayed	1	​
History of drug allergy^a^	62	24.3
History of multiple allergies (drug allergy, food allergy and/or respiratory allergy)	19	7.5
History of allergy to local anesthetics^b^	14	5.5
History of food allergy	2	0.8
Other risk factors	​	​
Cutaneous symptoms (Angioœdema, dermatitis, urticaria)	18	7.1
Respiratory symptoms (allergic rhinitis, asthma)	7	2.7
Family history of	​	​
Anaphylaxis to perioperative agents	3	1.2
Drugs allergy	1	0.4
Other history of allergy^c^	5	2.0
Concomitant allergy		
Drug allergy (excluding perioperative agents)	179	70.2
Respiratory allergy	136	53.3
Food allergy	65	25.5
Pet allergy	4	1.6
Allergy to other agents^d^	38	15.0
Smokers	82	32.2

^a^ Penicillins, cephalosporins, sulfonamides, fluoroquinolones, macrolides, chloramphenicol, nitroimidazole, non-steroidal anti-inflammatory drugs, hyoscine butylbromide, methylprednisolone, acetaminophen, aspirin, rabeprazole, iron, contrast agents.

^b^ At dentist or pre-botox.

^c^ Latex, botulinum toxin, nylon or adhesives.

^d^ Paraphenylenediamine, synthetic materials, adhesives, latex, botulinum toxin, polyethylene glycol, povidone-iodine, nickel, graphene oxide.

SPTs: skin prick tests, F: female, M: male.

**FIGURE 2:**
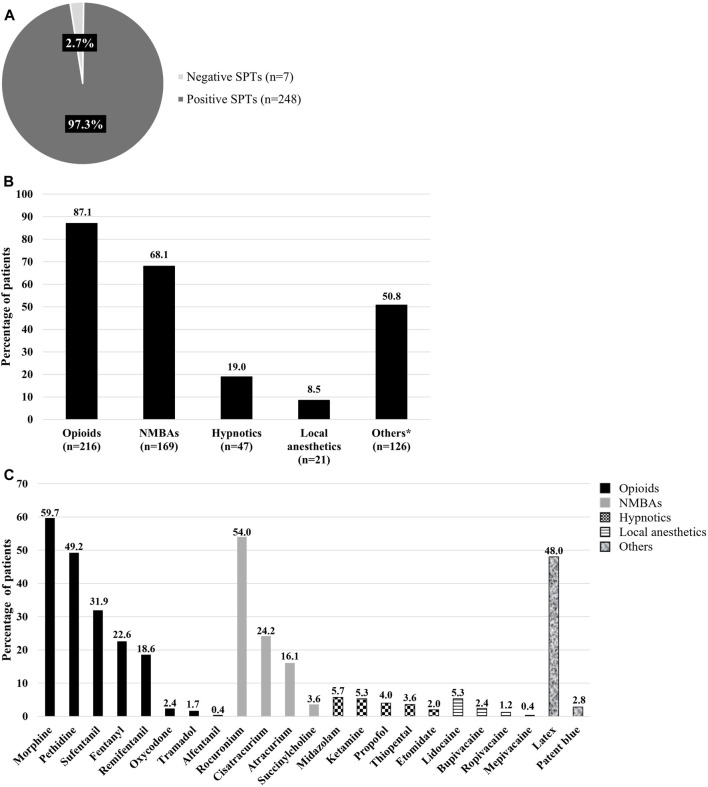
**(A)** Percentage of patients with positive or negative skin prick tests results among the 255 patients. **(B)** Percentage of positive results for the therapeutic class or agents obtained in patients with positive skin prick tests. *Others include latex and patent blue. **The percentages do not add up to 100 because a patient may be sensitized to one or more classes of perioperative agents. NMBAs: Neuromuscular blocking agents. **(C)** Percentage of patients with positive skin prick tests for each agent within the different therapeutic classes used in perioperative care. * The percentages do not add up to 100% because a patient may be sensitized to one or more molecules. 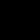 Opioids. 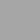 NMBAs. 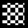 Hypnotics. 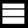 Local anesthetics. 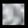 Others.

All 255 patients underwent SPTs for perioperative agents. Of these, 97.3% (n = 248) tested positive for one or more substances, while 2.7% (n = 7) tested negative, with no history of POH reactions among the latter group (Figure 2A). The results for the 248 patients who tested positive are as follows: 87.1% (n = 216) reacted to opioids, 68.1% (n = 169) to NMBAs, 19% (n = 47) to hypnotics, 8.5% (n = 21) to local anesthetics, and 50.8% (n = 126) to other substances such as latex and patent blue (Figure 2B).

We identified the most sensitizing molecules within each class of perioperative agents. Among opioids, the most common sensitizers were morphine (59.7%, n = 148), pethidine (49.2%, n = 122), sufentanil (31.9%, n = 79), fentanyl (22.6%, n = 56), and remifentanil (18.6%, n = 46), while oxycodone (2.4%, n = 6), tramadol (1.7%, n = 4), and alfentanil (0.4%, n = 1) were less commonly sensitizing. Within NMBAs class, rocuronium (54.0%, n = 134), cisatracurium (24.2%, n = 60), and atracurium (16.1%, n = 40) were the most frequent sensitizers, with succinylcholine sensitization being rare (3.6%, n = 9). For hypnotics, midazolam (5.7%, n = 14), ketamine (5.3%, n = 13), propofol (4.0%, n = 10), and thiopental (3.6%, n = 9) were the most sensitizing, while 2.0% (n = 5) were sensitized to etomidate. Additionally, 48.0% (n = 119) of patients were sensitized to latex and 2.8% (n = 7) to patent blue (Figure 2C).

Table 2 highlights the major co-sensitization identified among agents within the same therapeutic class of drugs used in the perioperative setting. Of the 216 patients with positive SPTs to opioids, 71 were sensitized to only one molecule, including 31 cases of morphine. Additionally, 74 patients showed co-sensitization to two opioid molecules, including 35 cases of morphine and pethidine, and 45 patients had co-sensitization to three molecules, including 9 cases of morphine, pethidine, and fentanyl. Among the 169 patients sensitized to NMBAs, 103 were exclusively sensitized to one molecule, including 73 cases of rocuronium, while 58 patients showed co-sensitization to two molecules, including 32 cases of rocuronium and cisatracurium. However, co-sensitization was relatively lower in the hypnotic and local anesthetic classes. Specifically, 3 out of 47 patients and 2 out of 21 patients showed co-sensitization in the hypnotic and local anesthetic classes, respectively. Moreover, 11 patients were sensitized only to midazolam or ketamine among the 44 cases identified for hypnotics, and 11 patients were sensitized to lidocaine among the 19 cases identified for local anesthetics.

**TABLE 2:** Most common associations evaluating possible co-sensitization to molecules in the same therapeutic class in patients with positive skin prick tests.

Class of perioperative agents	n	Numbre of molecules	n	Most frequent molecules^a^	n
Opioids	216	One molecule	71	Morphine	31
		Two molecules	74	Morphine, pethidine	35
		Three molecules	45	Morphine, pethidine, fentanyl	9
		Four molecules	22	Morphine, pethidine, fentanyl, sufentanil	9
		Five molecules	4	Morphine, pethidine, fentanyl, sufentanil, remifentanil	3
NMBAs	169	One molecule	103	Rocuronium	73
		Two molecules	58	Rocuronium, cisatracurium	32
		Three molecules	8	Rocuronium, cisatracurium, atracurium	6
Hypnotics	47	One molecule	44	Midazolam	11
		​	​	Ketamine	11
		Two molecules	2	Midazolam, ketamine	1
		Three molecules	1	Midazolam, ketamine, etomidate	1
Local anesthetics	21	One molecule Two molecules	19 2	Lidocaine	11
				Lidocaine, bupivacaine	1
				Lidocaine, ropivacaine	1

^a^ For each number of molecules, only the most frequent association of co-sensitization is described.

Table 3 highlights the most common co-sensitization patterns among perioperative agents. Key associations identified include “morphine and latex” (6 cases), “rocuronium, morphine, and pethidine” (7 cases), “rocuronium, morphine, and latex” (5 cases), and “rocuronium, morphine, pethidine, and latex” (4 cases).

**TABLE 3:** Most common associations evaluating possible co-sensitization among agents from various classes in patients with positive skin prick tests.

Number of molecules	Most frequent associations^a^	n
Two molecules	Morphine, latex	6
	Pethidine, latex	3
	Rocuronium, morphine	3
Three molecules	Rocuronium, morphine, pethidine	7
	Rocuronium, morphine, latex	5
	Morphine, pethidine, latex	3
	Rocuronium, cisatracurium, latex	3
Four molecules	Rocuronium, morphine, pethidine, latex	4

^a^ For each number of molecules, only the most frequent association of co-sensitization is described.

We focused on the 124 patients, out of a total of 255, who had a history of POH reactions. The majority of patients (80.6%) were women and 64.5% (80 patients) had a history of concomitant drug allergy. Generally, they had allergies to penicillin G, amoxicillin/clavulanic acid and NSAIDs, as reported in the anamnesis. SPTs assessing additional molecules were performed in 57 patients (46.0%) with a history of POH reactions, alongside SPTs for perioperatively used molecules. Among these 57 patients, 68.4% (39 patients) tested predominantly positive for penicillin G (14 cases) and the amoxicillin-clavulanic acid combination (12 cases) (data not shown).

**FIGURE 3:**
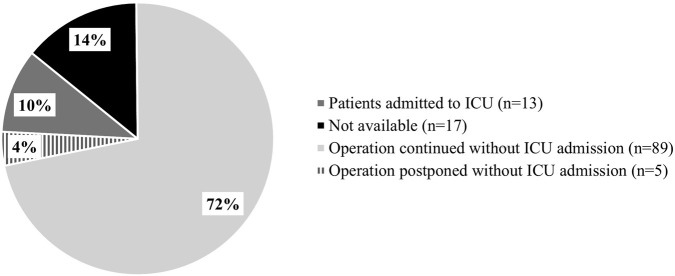
Patient Outcomes Following a Perioperative Hypersensitivity Reaction (n = 124, with a history of POH) ICU: Intensive care units.

**FIGURE 4:**
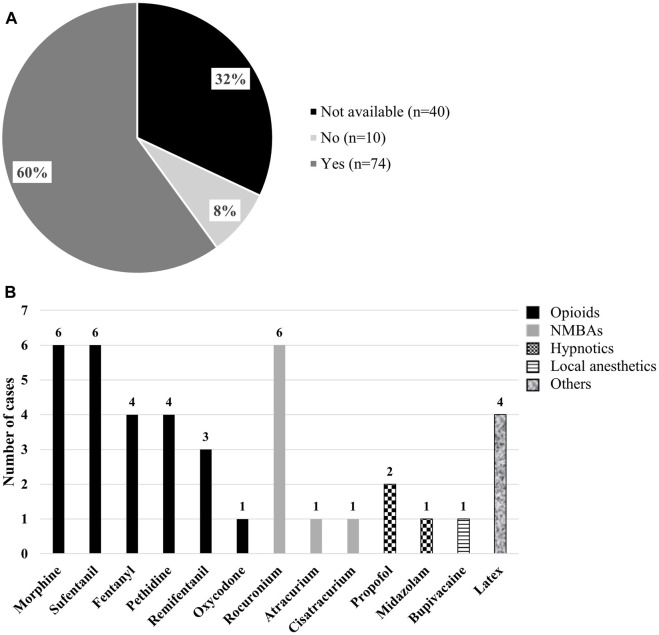
**(A)** Results of the 124 Patients with a history of perioperative hypersensitivity reactions, following previous surgery and exposure to perioperative agents. **(B)** Molecules tested positive by Skin Prick Tests in patients with perioperative hypersensitivity reactions, without previous surgery or exposure to perioperative agent.

Of the 124 patients, 99.2% (123 patients) experienced anaphylaxis, while only 0.8% (1 patient) had a delayed reaction with facial dermatitis and bullous lesions. Total IgE levels were assessed in 17 patients with a history of POH reaction; 14 patients reported high levels between 100 and 2190 IU/mL (Supplementary Table S2). Tryptase levels at the time of reaction were measured in 6 patients; 2 patients reported high levels between 30 and 36 μg/L (Supplementary Table S3). Anaphylactic reactions were classified using the Ring and Messmer system (Dewachter and Savic, 2019; Ring and Messmer, 1977): 33.1% (n = 41) were grade I, 16.9% (n = 21) were grade II, 45.2% (n = 56) were grade III, and 4.0% (n = 5) were grade IV (Table 4). Following POH reactions in the 124 patients, surgeries were continued in 72% (n = 89) of cases, postponed without intensive care unit (ICU) admission in 4% (n = 5), and required ICU admission in 10% (n = 13) (Figure 3). We then evaluated the surgical history of the patients. Among the 124 patients with a history of POH, 60% (n = 74) had undergone one or more surgeries prior to the procedure associated with the POH reaction, 8% had no prior surgeries (n = 10), and information on previous surgeries was missing for 32% (n = 40) of patients (Figure 4A). 10 patients (8%) of the 124 patients with POH had not been previously exposed to anesthetic products. Consequently, Figure 4B lists the molecules to which these patients were sensitized despite no prior exposure. The most frequently obtained molecules were morphine (6 cases), sufentanil (6 cases), and rocuronium (6 cases).

**TABLE 4:** Clinical manifestations of perioperative hypersensitivity reactions in 124 patients with a history of POH.

Symptoms	Immediate hypersensitivity n	%	Delayed hypersensitivity n	%
Anaphylaxis	123	99.2	0	0
Patients were classified according to Ring and Messmer grading system*	​	​	​	​
Grade I	41	33.1	​	​
Grade II	21	16.9	​	​
Grade III	56	45.2	​	​
Grade IV	5	4.0	​	​
*Face dermatitis and bullous lesions*	*0*	*0*	*1*	*0.8*

* Ring and Messmer grading system.

- Grade I: generalized mucocutaneous signs.

- Grade II: moderate multivisceral manifestations.

- Grade III: Severe life-threatening multivisceral manifestations.

- Grade IV: cardiopulmonary arrest.

We evaluated the culprit drugs involved in these 124 patients with a history of POH reactions. A relationship between the molecules used during these reactions and SPTs results was observed, with causative agents identified in 38 patients based on both anesthesia records and SPTs findings. Opioids (36.8%, n = 14) and NMBAs (23.7%, n = 9) were the most implicated culprit classes. Grade III anaphylaxis was predominantly manifested with NMBAs (66.7%, n = 6), while grade I anaphylaxis was largely manifested with opioids (42.9%, n = 6). In the NMBAs class, rocuronium was the most frequently identified (5 cases), followed by cisatracurium (2 cases), atracurium (1 case), and succinylcholine (1 case). In the opioids class, 8 cases involved morphine, 3 cases involved fentanyl, and 2 cases involved pethidine, with one case of co-sensitization between morphine and sufentanil. Latex was identified as the causative agent in two patients, one experiencing an immediate reaction and the other a delayed reaction, patent blue and lidocaine in two other patients and midazolam in one patient (Table 5 and complete data in Supplementary Table S4).

**TABLE 5:** Characteristics of the 38 patients with identified causative agents of perioperative hypersensitivity reactions.

Drug class	n (%)	Molecules	n	Symptoms and grading of perioperative hypersensitivity reactions n (%)
Opioids	14 (36.8)	Morphine Fentanyl Pethidine Morphine, sufentanil	8 3 2 1	Grade I 6 (42.9), grade II 2 (14.3), grade III 5 (35.7), grade IV 1 (7.1)
NMBAs	9 (23.7)	Rocuronium Cisatracurium Atracurium Succinylcholine	5 2 1 1	Grade I 1 (11.1), grade II 1 (11.1), grade III 6 (66.7), grade IV 1 (11.1)
Others	4 (10.5)	Latex Patent blue	2 2	Grade I 2 (50.0), grade III 1 (25.0), face dermatitis and bullous lesions with latex 1 (25.0)
Local anesthetics	2 (5.3)	Lidocaïne	2	Grade I 1 (50.0), grade II 1 (50.0)
Hypnotics	1 (2.6)	Midazolam	1	Grade I 1 (100.0)
Opioids, NMBAs	3 (7.9)	Fentanyl, rocuronium Sufentanil, cisatracurium	2 1	Grade III 3 (100.0)
Opioids, latex	2 (5.3)	Morphine, latex Sufentanil, latex	1 1	Grade I 2 (100.0)
Opioids, hypnotics	1 (2.6)	Fentanyl, propofol	1	Grade III 1 (100.0)
Opioids, NMBAs, hypnotics	1 (2.6)	Fentanyl, rocuronium, propofol	1	Grade II 1 (100.0)
Opioids, NMBAs, ondansetron	1 (2.6)	Fentanyl, remifentanil, rocuronium, ondansetron	1	Grade III 1 (100.0)

NMBAs: Neuromuscular blocking agents.

## Discussion

Epidemiological studies on POH reactions show variable outcomes, reflecting differences in study populations and clinical practices, with limited data from the Middle East (Beyaz et al., 2022; Kosciuczuk and Knapp, 2021; Yegin et al., 2025; Al-Ahmad et al., 2021). We previously published studies in 2014 and 2023, analyzing anesthetic allergies and drug hypersensitivity with positive SPTs to perioperative molecules (Irani, 2014; Dagher et al., 2024). This five-year retrospective study, conducted in a tertiary center, offers an in-depth analysis of POH patient profiles, with a focus on SPTs evaluation.

Among 255 patients referred for SPTs for various perioperative agents, the median age was 42 years and 84% were female, reflecting trends in most published studies (Beyaz et al., 2022; Moreau et al., 2024). Female predominance may be linked to higher and repeated drug consumption by women, exposure to cosmetics and household products containing a quaternary ammonium group, which could explain NMBAs sensitizations, as well as genetic, epigenetic, and hormonal factors influencing immune response (Pitlick and Volcheck, 2022; Salvati et al., 2019; Malvik et al., 2022). In our study, the most common reasons for consultation were a history of POH reactions, particularly of the immediate type (48.2%), and a history of drug allergies (24.3%). In daily practice, atopic patients and those with other drug allergies are often referred to allergy clinics for evaluation of possible general anesthetic allergy, despite this not being recommended in recent guidelines (Garvey et al., 2019).

In our study and among the 255 patients, 97.3% (n = 248) had positive SPT results for one or more substances. It should be noted that in our study intradermal and/or exposure tests were not performed. Drugs with a positive SPT were avoided, while those with a negative SPT were tolerated upon re-exposure. The high SPTs positivity rate reflects the study population’s characteristics, including prior POH reactions, or predisposing factors such as concomitant drug allergies, food allergies or multiple allergies. These factors have been consistently associated with an increased risk of POH (Zhang et al., 2024; Mirone et al., 2015), and our findings support their clinical relevance, highlighting the need for careful perioperative monitoring in patients presenting with these risk factors. The most common sensitizations were to opioids (87.1%), followed by NMBAs (68.1%). These findings are consistent with two Lebanese studies, which similarly reported high sensitization to opioids (94.1% and 82.2%) and NMBAs (47.1% and 63.3%) (Irani, 2014; Dagher et al., 2024).

For opioids, the percentage of cases is higher than what is typically described (Kosciuczuk and Knapp, 2021; Tacquard et al., 2024; Baldo, 2023). In our study, patients sensitized to opioids reacted to morphine (59.7%) and pethidine (49.2%). IgE-mediated hypersensitivity to opiates and semisynthetic opioids is rare and most POH reactions to morphine or pethidine result from nonspecific mast cell activation via MRGPRX2 (Garvey et al., 2019; Baldo and Pham, 2023). SPT results to opioids should be interpreted with caution, and avoidance of opioids based solely on SPT positivity may lead to unnecessary restriction. While opioids-specific IgE assays have limited application, there is a growing need for studies evaluating BAT in opioid allergy, as BAT can help identify MRGPRX2-mediated reactions, particularly when skin tests are positive but BAT is negative (Kalangara et al., 2019). Drug provocation test (DPT) remains the gold standard for confirming true clinical reactivity when performed after careful risk stratification and using individualized protocols (Barbaud et al., 2024; Mayorga et al., 2019). The importance of performing DPT in workup of opioid allergy was highlighted in a retrospective cohort of 98 patients referred for suspected opioid hypersensitivity. In this study, only 15% were confirmed with opioid allergy, highlighting the risk of overdiagnosis in the absence of confirmatory testing (Li et al., 2017). Chalhoub et al. reported that opioids are frequently prescribed in Lebanon, with codeine available over the counter (Chalhoub et al., 2021). Co-sensitization between morphine and codeine (both phenanthrenes) may contribute to the high morphine sensitization observed in the Lebanese population (Garvey et al., 2019; Van Cuilenborg et al., 2021). Co-sensitization among opioids observed in our study—such as morphine, pethidine, and fentanyl—can be attributed to structural similarities. Morphine and pethidine share a phenylpiperidine core and a phenylpropylamine group. Fentanyl and its derivatives (remifentanil, and sufentanil) possess a 4-anilidophenylpiperidine and 4-anilidophenylpropylamine structures which also share conformational similarities to morphine and pethidine, contributing to potential co-sensitization (Baldo and Pham, 2012).

In our study, rocuronium was the most common NMBA found positive by SPTs (54.0%), consistent with previous studies (Pirson et al., 2018; Irani, 2014; Mirone et al., 2015; Tacquard et al., 2024). NMBAs, including depolarizing (e.g., suxamethonium) and nondepolarizing agents (e.g., rocuronium, atracurium, cisatracurium), have seen a shift in usage, with increasing incidents of POH reactions to nondepolarizing agents like atracurium, rocuronium and cisatracurium which aligns with our findings (Warr et al., 2011; Mertes et al., 2011). The incidence of co-sensitization among NMBAs detected by SPTs is known to be frequent (60%–84%), due to shared antigenic quaternary ammonium groups (Peyneau et al., 2025; Molero Díez et al., 2023). Rocuronium and cisatracurium were the agents with the highest co-sensitization in our findings, although variability in co-sensitization patterns has been noted across regions, potentially reflecting difference in NMBAs usage (Tacquard et al., 2024; Molero Díez et al., 2023; Li et al., 2019; Sadleir et al., 2013). In published studies by Sadleir et al., Li et al. and Tacquard et al., only 5%, 6% and 12% of patients with rocuronium allergy, respectively, experienced co-sensitization to cisatracurium (Tacquard et al., 2024; Li et al., 2019; Sadleir et al., 2013).

In contrast to published data, where hypnotics accounted for between 2% and 10.4% of POH cases, our study found that hypnotics were responsible for 19% of POH reactions (Tacquard et al., 2024; Baldo, 2023). According to our findings, midazolam, ketamine, propofol, thiopental, and etomidate were implicated, with midazolam being the most frequently involved. In our study, 4% of patients tested positive by SPT for propofol, which is higher than the rates typically reported, namely 1.3% and 0.65% of POH reactions in France and Australia, respectively (Baldo, 2023). The clinical relevance of a positive SPT to propofol cannot be definitively established in the absence of intradermal testing or controlled provocation testing. Drugs such as propofol, demand special care to be taken in planning the provocations and is not feasible in our outpatient clinic setting (Garvey et al., 2019; Broyles et al., 2020). In our study, amide local anesthetics (lidocaine, bupivacaine, ropivacaine, and mepivacaine) accounted for 8.5% of POH cases, with lidocaine being the most frequently implicated. Although amide local anesthetics are generally considered less allergenic than esters, our findings revealed a higher prevalence of hypersensitivity reactions than previously reported, likely reflecting their predominant use in clinical practice (Carrión Sari et al., 2021; Bhole et al., 2012). Patent blue is a dye used in detecting sentinel lymph nodes during breast cancer diagnosis. It has been linked to 5%–6% of POH cases in the UK and France, our study found similar incidence with patent blue implicated in 2.8% of cases (Van De Ven et al., 2022).

Furthermore, 48.0% of patients in our study were latex sensitized. SPTs and specific IgE are considered the most reliable methods of the detection of latex allergy (Bernardini et al., 2008). Latex allergy incidence has declined in several countries, from 20% to 5% in France (1997–2012), with similar decreases in Spain and the USA, and no cases reported in the UK (Ahmed and Savic, 2020). Latex sensitization and allergy are closely related to exposure levels, with the highest risk in individuals with occupational exposure (e.g., healthcare workers) or repeated medical procedures, particularly patients with more than five surgeries, repeated anesthesia, or frequent catheterizations (Arasi et al., 2023; Claudio et al., 2016). The high prevalence of latex sensitization in Lebanon underscores the need to limit the use of latex-based products in healthcare settings, a strategy shown to effectively reduce new sensitizations (Turjanmaa et al., 2002). Hypersensitivity reactions to chlorhexidine are increasingly reported in some countries (Broyles et al., 2020; Jagadish et al., 2026). Although SPTs were performed for commonly implicated perioperative drugs, antiseptics such as chlorhexidine and povidone-iodine were not systematically tested. This may have underestimated their role in perioperative anaphylaxis and warrants evaluation in future studies.

A specific causal agent could be identified in 38 patients based on both anesthesia records and SPTs findings, highlighting a relationship between the molecules used during these reactions and SPTs results. However, the absence of specific serum IgE testing in Lebanon limits further confirmation. This approach enabled us to identify a specific causal agent in 38 patients and suggest alternative perioperative options, with opioids (36.8%) and NMBAs (23.7%) being the most implicated culprit classes. Moreover, among patients with no prior surgeries, morphine, sufentanil, and rocuronium were identified as causative agents for POH. Reactions can occur even in patients receiving these drugs for the first time, and they may be due to direct mast cell degranulation. In the case of NMBAs, cross-reactivity with substances containing quaternary ammonium groups such as cosmetics, foods, industrial materials, and disinfectants may also contribute to these reactions (Banerji et al., 2021; Peyneau et al., 2022). Tryptase levels during the reaction were measured in six patients. We propose that follow-up measurement of baseline serum tryptase may enhance diagnostic accuracy by detecting systemic mastocytosis as a potential underlying cause of severe anaphylaxis. In conclusion, the study highlighted patient profiles and common agents involved in POH in a Lebanese population, and proved that SPTs are useful in identifying causes allowing to suggest safe alternatives. This evaluation is essential to minimize the risk of hypersensitivity reactions and to support both anesthesiologists and patients in making informed and safer perioperative decisions. Our findings highlight the need for national prospective data collection on POH, as established in other countries (Mertes et al., 2011; Harper et al., 2018; Zhang et al., 2022), and implementing more precise *ex vivo* tests, such as circulating specific IgE and BAT in reference laboratories, would improve the management of patients experiencing POH reactions in Lebanon.

## Data Availability

The data analyzed in this study is subject to the following licenses/restrictions: The datasets generated and/or analyzed during the current study are not publicly available due to patient confidentiality. Requests to access these datasets should be directed to iranica@yahoo.com or diane.antonios@usj.edu.lb.

## References

[B1] Ahmed S. Savic L. (2020). Latex: a rare but important cause of perioperative allergic reactions. BJA Educ. 20 (12), 398–399. 10.1016/j.bjae.2020.08.002 33456924 PMC7807988

[B2] Al-Ahmad M. Edin J. Musa F. Rodriguez-Bouza T. (2021). Drug allergy profile from a national drug allergy registry. Front. Pharmacol. 11, 555666. 10.3389/fphar.2020.555666 33542684 PMC7851708

[B3] Arasi S. Barni S. Caminiti L. Castagnoli R. Giovannini M. Liotti L. (2023). Latex allergy in children. J. Clin. Med. 13 (1), 124. 10.3390/jcm13010124 38202131 PMC10779698

[B4] Baldo B. A. (2023). Allergic and other adverse reactions to drugs used in anesthesia and surgery. Anesthesiol. Perioper. Sci. 1 (2), 16. 10.1007/s44254-023-00018-2 40476920 PMC10264870

[B5] Baldo B. A. Pham N. H. (2012). Histamine-releasing and allergenic properties of opioid analgesic drugs: resolving the two. Anaesth. Intensive Care 40 (2), 216–235. 10.1177/0310057X1204000204 22417016

[B6] Baldo B. A. Pham N. H. (2023). Opioid toxicity: histamine, hypersensitivity, and MRGPRX2. Arch. Toxicol. 97 (2), 359–375. 10.1007/s00204-022-03402-2 36344690

[B7] Banerji A. Bhattacharya G. Huebner E. Fu X. Camargo C. A. Guyer A. (2021). Perioperative allergic reactions: allergy assessment and subsequent Anesthesia. J. Allergy Clin. Immunol. Pract. 9 (5), 1980–1991. 10.1016/j.jaip.2020.11.025 33248280

[B8] Barbaud A. Garvey L. H. Torres M. Laguna J. J. Arcolaci A. Bonadonna P. (2024). EAACI/ENDA position paper on drug provocation testing. Allergy 79 (3), 565–579. 10.1111/all.15996 38155501

[B9] Bernardini R. Pucci N. Azzari C. Novembre E. De Martino M. Milani M. (2008). Sensitivity and specificity of different skin prick tests with latex extracts in pediatric patients with suspected natural rubber latex allergy – a cohort study. Pediatr. Allergy Immunol. 19 (4), 315–318. 10.1111/j.1399-3038.2007.00662.x 18266828

[B10] Beyaz S. Coskun R. Oztop N. Aygun E. Sungur M. O. Seyhan T. O. (2022). Evaluation of skin test indications for general anesthetics in real life: a prospective cohort study. Braz J. Anesthesiol. Engl. Ed. 72 (3), 350–358. 10.1016/j.bjane.2021.07.005 34324936 PMC9373089

[B11] Bhole M. V. Manson A. L. Seneviratne S. L. Misbah S. A. (2012). IgE-mediated allergy to local anaesthetics: separating fact from perception: a UK perspective. Br. J. Anaesth. 108 (6), 903–911. 10.1093/bja/aes162 22593127

[B12] Broyles A. D. Banerji A. Barmettler S. Biggs C. M. Blumenthal K. Brennan P. J. (2020). Practical guidance for the evaluation and management of drug hypersensitivity: specific drugs. J. Allergy Clin. Immunol. Pract. 8 (9), S16–S116. 10.1016/j.jaip.2020.08.006 33039007

[B13] Bruhns P. Chollet-Martin S. (2021). Mechanisms of human drug-induced anaphylaxis. J. Allergy Clin. Immunol. 147 (4), 1133–1142. 10.1016/j.jaci.2021.02.013 33832695

[B14] Carrión Sari S. Lezaun A. Colas Sanz C. (2021). Anaphylaxis due to perioperative intravenous lidocaine. J. Investig. Allergol. Clin. Immunol. 31 (2), 164–165. 10.18176/jiaci.0626 32573457

[B15] Chalhoub C. Obeid S. Hallit R. Salameh P. Hallit S. (2021). Addictive profiles of Lebanese university students in terms of smoking, alcohol, and illegal drug use. Environ. Sci. Pollut. Res. 28 (41), 57657–57666. 10.1007/s11356-021-14751-3 34091844 PMC8179089

[B16] Claudio A. Natalia A. Julio N. (2016). Prevalence of latex allergy in a population of patients diagnosed with myelomeningocele. Arch. Argent. Pediatr. 114 (1), 30–35. 10.5546/aap.2016.eng.30 26914072

[B17] Dagher J. Antonios D. Chollet-Martin S. De Chaisemartin L. Pallardy M. Azouri H. (2024). Drug-induced hypersensitivity reactions in a Lebanese outpatient population: a decade-long retrospective analysis (2012-2021). J. Allergy Clin. Immunol. Glob. 3 (1), 100169. 10.1016/j.jacig.2023.100169 37876854 PMC10590748

[B18] Dewachter P. Savic L. (2019). Perioperative anaphylaxis: pathophysiology, clinical presentation and management. BJA Educ. 19 (10), 313–320. 10.1016/j.bjae.2019.06.002 33456852 PMC7807982

[B19] Ebo D. G. Clarke R. C. Mertes P. M. Platt P. R. Sabato V. Sadleir P. H. M. (2019). Molecular mechanisms and pathophysiology of perioperative hypersensitivity and anaphylaxis: a narrative review. Br. J. Anaesth. 123 (1), e38–e49. 10.1016/j.bja.2019.01.031 30916022

[B20] Garvey L. H. (2016). Perioperative hypersensitivity reactions: diagnosis, treatment and evaluation. Curr. Treat. Options Allergy 3 (2), 113–128. 10.1007/s40521-016-0078-0

[B21] Garvey L. H. Ebo D. G. Mertes P. Dewachter P. Garcez T. Kopac P. (2019). An EAACI position paper on the investigation of perioperative immediate hypersensitivity reactions. Allergy 74 (10), 1872–1884. 10.1111/all.13820 30964555

[B22] Harper N. J. N. Cook T. M. Garcez T. Farmer L. Floss K. Marinho S. (2018). Anaesthesia, surgery, and life-threatening allergic reactions: epidemiology and clinical features of perioperative anaphylaxis in the 6th National Audit Project (NAP6). Br. J. Anaesth. 121 (1), 159–171. 10.1016/j.bja.2018.04.014 29935567

[B23] Irani C. (2014). Allergy to general anesthetics: evaluation of patients profile. Int. J. Anesth. Anesthesiol. 1 (3). 10.23937/2377-4630/1/3/1013

[B24] Jagadish I. Estrada-Mendizabal R. J. Gonzalez-Estrada A. Volcheck G. W. Rukasin C. R. F. (2026). A 20-year FAERS analysis of hypersensitivity reports to chlorhexidine and povidone-iodine (2004-2024). J. Allergy Clin. Immunol. Glob. 5 (1), 100611. 10.1016/j.jacig.2025.100611 41459439 PMC12743433

[B25] Jönsson F. De Chaisemartin L. Granger V. Gouel-Chéron A. Gillis C. M. Zhu Q. (2019). An IgG-induced neutrophil activation pathway contributes to human drug-induced anaphylaxis. Sci. Transl. Med. 11 (500), eaat1479. 10.1126/scitranslmed.aat1479 31292264

[B26] Kalangara J. Potru S. Kuruvilla M. (2019). Clinical manifestations and diagnostic evaluation of opioid allergy labels – a review. J. Pain Palliat. Care Pharmacother. 33 (3–4), 131–140. 10.1080/15360288.2019.1666955 31638447

[B27] Kosciuczuk U. Knapp P. (2021). What do we know about perioperative hypersensitivity reactions and what can we do to improve perioperative safety? Ann. Med. 53 (1), 1772–1778. 10.1080/07853890.2021.1976818 34632895 PMC8510593

[B28] Li P. H. Ue K. L. Wagner A. Rutkowski R. Rutkowski K. (2017). Opioid hypersensitivity: predictors of allergy and role of drug provocation testing. J. Allergy Clin. Immunol. Pract. 5 (6), 1601–1606. 10.1016/j.jaip.2017.03.035 28550985

[B29] Li J. Best O. G. Rose M. A. Green S. L. Fulton R. B. Capon M. J. (2019). Assessing cross-reactivity to neuromuscular blocking agents by skin and basophil activation tests in patients with neuromuscular blocking agent anaphylaxis. Br. J. Anaesth. 123 (1), e144–e150. 10.1016/j.bja.2019.03.001 30961915

[B30] Malvik L. B. De Pater G. H. Dahle G. O. Guttormsen A. B. (2022). Gender‐specific decline in perioperative allergic reactions in Norway after withdrawal of pholcodine. Allergy 77 (4), 1317–1319. 10.1111/all.15201 34963030

[B31] Mayorga C. Ebo D. G. Lang D. M. Pichler W. J. Sabato V. Park M. A. (2019). Controversies in drug allergy: *in vitro* testing. J. Allergy Clin. Immunol. 143 (1), 56–65. 10.1016/j.jaci.2018.09.022 30573343

[B32] Mertes P. M. Alla F. Tréchot P. Auroy Y. Jougla E (2011). Anaphylaxis during anesthesia in France: an 8-year national survey. J. Allergy Clin. Immunol. 128 (2), 366–373. 10.1016/j.jaci.2011.03.003 21497888

[B33] Mirone C. Preziosi D. Mascheri A. Micarelli G. Farioli L. Balossi L. G. (2015). Identification of risk factors of severe hypersensitivity reactions in general anaesthesia. Clin. Mol. Allergy CMA 13 (1), 11. 10.1186/s12948-015-0017-9 26101469 PMC4476085

[B34] Molero Díez Y. B. Sanchis Dux R. Sánchez Tabernero Á. De Diego Fernández A. (2023). Anaphylaxis to neuromuscular blocking agents: cross-reactivity between rocuronium and cisatracurium. Can. J. Anesth. Can. Anesth. 70 (2), 286–287. 10.1007/s12630-022-02375-1 36509951

[B35] Moreau A. Gouel-Chéron A. Roland E. McGee K. Plaud B. Blet A. (2023). Allergie peranesthésique: revue et guide de bonnes pratiques. Anesth. Réanimation 9 (2), 184–196. 10.1016/j.anrea.2023.01.013

[B36] Moreau A. Chérifa M. Roland E. Mc Gee K. Plaud B. Gouel-Cheron A. (2024). Exploring the landscape of perioperative immediate hypersensitivity: a comprehensive 6-Year monocentric observational analysis on epidemiology and risk factors. Int. Arch. Allergy Immunol. 186, 1–5. 10.1159/000542734 39571554

[B37] Peyneau M. De Chaisemartin L. Gigant N. Chollet-Martin S. Kerdine-Römer S. (2022). Quaternary ammonium compounds in hypersensitivity reactions. Front. Toxicol. 4, 973680. 10.3389/ftox.2022.973680 36211198 PMC9534575

[B38] Peyneau M. Zeller M. Paulet V. Noël B. Damiens M. H. Szely N. (2025). Quaternary ammoniums activate human dendritic cells and induce a specific T-cell response *in vitro* . Allergol. Int. 74 (1), 105–114. 10.1016/j.alit.2024.07.003 39237430

[B39] Pirson F. Kremer Y. Darchambeau L. (2018). Accidents d’hypersensibilité per-anesthésiques: expérience d’un hôpital universitaire belge. Rev. Fr. Allergol. 58 (8), 564–573. 10.1016/j.reval.2018.09.010

[B40] Pitlick M. M. Volcheck G. W. (2022). Perioperative anaphylaxis. Immunol. Allergy Clin. North Am. 42 (1), 145–159. 10.1016/j.iac.2021.09.002 34823744

[B41] Ring J. Messmer K. (1977). Incidence and severity of anaphylactoid reactions to colloid volume substitutes. Lancet 309 (8009), 466–469. 10.1016/S0140-6736(77)91953-5 65572

[B42] Sabato V. Platt P. Garcez T. Cooke P. (2019). Suspected perioperative allergic reactions: nomenclature and terminology. Br. J. Anaesth. 123 (1), e13–e15. 10.1016/j.bja.2019.05.001 31126621

[B43] Sadleir P. H. M. Clarke R. C. Bunning D. L. Platt P. R. (2013). Anaphylaxis to neuromuscular blocking drugs: incidence and cross-reactivity in Western Australia from 2002 to 2011. Br. J. Anaesth. 110 (6), 981–987. 10.1093/bja/aes506 23335568

[B44] Salik I. Hernandez J. (2019). Gadolinium induced anaphylaxis under general anesthesia in a patient with sickle cell disease. J. Clin. Anesth. 54, 47. 10.1016/j.jclinane.2018.10.048 30391454

[B45] Salvati L. Vitiello G. Parronchi P. (2019). Gender differences in anaphylaxis. Curr. Opin. Allergy Clin. Immunol. 19 (5), 417–424. 10.1097/ACI.0000000000000568 31465313

[B46] Tacquard C. Iba T. Levy J. H. (2023). Perioperative-anaphylaxis. Anesthesiology 138 (1), 100–110. 10.1097/ALN.0000000000004419 36413685

[B47] Tacquard C. Serrier J. Viville S. Chiriac A. M. Franchina S. Gouel-Cheron A. (2024). Epidemiology of perioperative anaphylaxis in France in 2017–2018: the 11th GERAP survey. Br. J. Anaesth. 132 (6), 1230–1237. 10.1016/j.bja.2024.01.044 38493055 PMC11130666

[B48] Thanachit K. Chamard W. Torpong T. (2024). Perioperative immediate hypersensitivity incidence, clinical characteristics, and outcomes after allergological evaluation: a multi-disciplinary protocol from tertiary hospital, Thailand. Asian Pac J. Allergy Immunol. 10.12932/AP-150922-1456 36773280

[B49] Turjanmaa K. Alenius H. Reunala T. Palosuo T. (2002). Recent developments in latex allergy. Curr. Opin. Allergy Clin. Immunol. 2 (5), 407–412. 10.1097/00130832-200210000-00007 12582324

[B50] Van Cuilenborg V. R. Hermanides J. Bos E. M. E. Hollmann M. W. Preckel B. Kooij F. O. (2021). Perioperative approach of allergic patients. Best. Pract. Res. Clin. Anaesthesiol. 35 (1), 11–25. 10.1016/j.bpa.2020.03.003 33742571

[B51] Van De Ven AAJM Oude Elberink J. N. G. Nederhoed V. Van Maaren M. S. Tupker R. Röckmann‐Helmbach H. (2022). Causes of perioperative hypersensitivity reactions in the Netherlands from 2002 to 2014. Clin. Exp. Allergy 52 (1), 192–196. 10.1111/cea.14042 34741764 PMC9298996

[B52] Volcheck G. W. Mertes P. M. (2014). Local and general anesthetics immediate hypersensitivity reactions. Immunol. Allergy Clin. North Am. 34 (3), 525–546. 10.1016/j.iac.2014.03.004 25017676

[B53] Volcheck G. W. Melchiors B. B. Farooque S. Gonzalez-Estrada A. Mertes P. M. Savic L. (2023). Perioperative hypersensitivity evaluation and management: a practical approach. J. Allergy Clin. Immunol. Pract. 11 (2), 382–392. 10.1016/j.jaip.2022.11.012 36436761

[B54] Warr J. Thiboutot Z. Rose L. Mehta S. Burry L. D. (2011). Current therapeutic uses, pharmacology, and clinical considerations of neuromuscular blocking agents for critically III adults. Ann. Pharmacother. 45 (9), 1116–1126. 10.1345/aph.1Q004 21828347

[B55] Yegin K. Z. Bulut İ. Saydın F. Yavuz D. Cosar N. D. Katran M. (2025). The cause of perioperative hypersensitivity in adults and consequences of subsequent anesthesia. Allergol. Immunopathol. Madr. 53 (2), 113–123. 10.15586/aei.v53i2.1281 40088030

[B56] Zhang P. Liu X. Li W. Gong R. Zuo J. Sun R. (2022). Epidemiology of suspected life-threatening perioperative anaphylaxis: a cross-sectional multicentre study in China. Br. J. Anaesth. 128 (1), 45–54. 10.1016/j.bja.2021.09.020 34742540

[B57] Zhang P. Wan Y. Li H. Lin X. (2024). Relationship between perioperative anaphylaxis and history of allergies or allergic diseases: a systematic review and meta-analysis with meta-regression. J. Clin. Anesth. 94, 111408. 10.1016/j.jclinane.2024.111408 38387242

