# Supplementary material for: Perioperative hypersensitivity reactions: a retrospective study (2018–2023) in a Lebanese tertiary clinic
Source: Front Pharmacol. 2026 Mar 25;17:1767932. doi: 10.3389/fphar.2026.1767932 (PMC13056635; doi:10.3389/fphar.2026.1767932)
Supplement: Supplementary file 1 [file Table1.docx]

**Supplementary material**

**Supplementary Table 1- Drug concentrations used for skin prick testing**

| **Class/drugs** | **Maximum non irritative concentration SPT** | **References** |
| --- | --- | --- |
| **Opioids** |  |  |
| Morphine | 1 mg/ml | 25,48 |
| Pethidine | 5 mg/ml | Clinical practice |
| Sufentanil | 0,005 mg/ml | 25,48 |
| Fentanyl | 0,05 mg/ml | 25,48 |
| Remifentanil | 0,05 mg/ml | 25,48 |
| Oxycodone | 1mg/ml | Clinical practice |
| Tramadol | 5mg/ml | Clinical practice |
| Alfentanil | 0.5 mg/ml | 25,48 |
| **NMBAs** |  |  |
| Rocuronium | 10 mg/ml | 25,48 |
| Cisatracurium | 2 mg/ml | 25,48 |
| Atracurium | 1 mg/ml | 25,48 |
| Succinylcholine | 10 mg/ml | 25,48 |
| **Hypnotics** |  |  |
| Midazolam | 5 mg/ml | 25,48 |
| Ketamine | 10 mg/ml | 48 |
| Propofol | 10 mg/ml | 25,48 |
| Thiopental | 25 mg/ml | 25 |
| Etomidate | 2 mg/ml | 25,48 |
| **Local anesthetics** |  |  |
| Lidocaine | 10 mg/ml | 25,48 |
| Bupivacaine | 2,5 mg/ml | 25,48 |
| Ropivacaine | 10 mg/ml | 25 |
| Mepivacaine | 10 mg/ml | 48 |
| **Others** |  |  |
| Latex | Latex extract from Diater^®^ |  |
| Patent blue | 25 mg/ml | 25 |

**Supplementary table II: Total IgE levels assessed in 17 patients with a history of perioperative hypersensitivity reactions**

| **Patients with a history of POH reaction (n=124)** | | |
| --- | --- | --- |
| **Total IgE level (UI/ mL)**  **(n=17)** | **<100**  **(n=3)** | **>100**  **(n=14)** |
|  | 7.2 | 100 |
|  | 36 | 111 |
|  | 76 | 127 |
|  |  | 138 |
|  |  | 154 |
|  |  | 165 |
|  |  | 199 |
|  |  | 337 |
|  |  | 443 |
|  |  | 570 |
|  |  | 641 |
|  |  | 1133 |
|  |  | 2190 |

**Supplementary table III: Tryptase levels assessed in 6 patients with a history of perioperative hypersensitivity reactions**

| **Patients with a history of POH reaction (n=124)** | | |
| --- | --- | --- |
| **Tryptase level (μg/ mL)**  **(n=6)** | **< 11.4 μg/ L**  **(n=4)** | **> 11.4 μg/ L**  **(n=2)** |
|  | 2.5 | 30 |
|  | 3.9 | 36 |
|  | 3.9 |  |
|  | 7.8 |  |

**Supplementary table IV: Complete data of 38 patients with identified causative agents of perioperative hypersensitivity reactions, including grading, pre-exposure to perioperative agents, IgE and tryptase levels, and other positive molecules (NA= Not available).**

| **Drugs class** | **Identified Molecule (s)** | **Grade** | **Pre-exposure** | **Total IgE** | **Tryptase** | **Positive skin prick tests for other molecules** |
| --- | --- | --- | --- | --- | --- | --- |
| **Opioids** | Morphine | 1 | NA | NA | NA | Cephalosporin |
|  | Morphine | 1 | NA | NA | NA |  |
|  | Morphine | 1 | YES | NA | NA |  |
|  | Morphine | 1 | NA | NA | NA |  |
|  | Morphine | 2 | YES | NA | NA |  |
|  | Morphine | 2 | NA | NA | NA | Penicillin/ Cephalosporin |
|  | Morphine | 3 | YES | NA | NA | Clindamycin/ Ciprofloxacin |
|  | Morphine | 3 | YES | NA | NA |  |
|  | Fentanyl | 3 | NA | 165 | 2.5 (Low) |  |
|  | Fentanyl | 3 | YES | NA | NA | Penicillin |
|  | Fentanyl | 3 | YES | NA | NA |  |
|  | Pethidine | 1 | YES | NA | NA |  |
|  | Pethidine | 4 | NO | NA | NA | Amoxicllin Clavulanic acid |
|  | Morphine, Sufentanil | 1 | YES | NA | NA |  |
| **NMBAs** | Rocuronium | 2 | NA | NA | 7.8 (Low) |  |
|  | Rocuronium | 3 | NA | NA | NA | Penicillin |
|  | Rocuronium | 3 | NA | NA | NA | Amoxicllin Clavulanic acid |
|  | Rocuronium | 3 | NA | NA | NA |  |
|  | Rocuronium | 4 | YES | 199 | NA |  |
|  | Cisatracurium | 3 | NA | NA | NA |  |
|  | Cisatracurium | 3 | YES | NA | NA |  |
|  | Succinylcholine | 1 | YES | 100 | NA |  |
|  | Atracurium | 3 | YES | NA | NA |  |
| **Others** | Latex | 1 | NA | NA | NA |  |
|  | Latex | Face dermatitis and bullous lesions | YES | NA | NA | Amoxicllin Clavulanic acid |
|  | Patent blue | 4 | NA | NA | NA |  |
| **Local anesthetics** | Lidocaine | 1 | YES | NA | NA |  |
|  | Lidocaine | 2 | NA | NA | NA | Cephalosporin |
| **Hypnotics** | Midazolam | 1 | NA | NA | NA |  |
| **Opioids + NMBAs** | Sufentanil/ Cisatracurium | 3 | YES | NA | NA |  |
|  | Fentanyl/ Rocuronium | 3 | YES | NA | NA |  |
|  | Fentanyl/ Rocuronium | 3 | NO | 641 | 30 (High) | Amoxicllin Clavulanic acid |
| **Opioids + Latex** | Sufentanil/ Latex | 1 | YES | NA | NA |  |
|  | Morphine/ Latex | 1 | YES | NA | NA |  |
| **Opioids + Hypnotics** | Fentanyl/ Propofol | 3 | YES | 154 | 3.9 (Low) |  |
| **Opioids + Hypnotics + NMBAs** | Fentanyl/ Propofol/ Rocuronium | 2 | NO | 570 | NA |  |
| **Opioids + NMBAs + Ondansetron** | Fentanyl/ Remifentanil/ Rocuronium/ Ondansetron | 3 | YES | NA | NA | Ondansetron |
